# Recent Advances in Treatment of Primary Central Nervous System Lymphoma

**DOI:** 10.1007/s11864-013-0252-6

**Published:** 2013-08-11

**Authors:** Lakshmi Nayak, Tracy T. Batchelor

**Affiliations:** 1 Dana-Farber Cancer Institute, Center for Neuro-Oncology, Department of Neurology, Brigham and Women’s Hospital, Harvard Medical School, 450 Brookline Avenue, Boston, MA 02215 USA; 2 Stephen E. and Catherine Pappas Center for Neuro-Oncology, Division of Hematology and Oncology, Department of Neurology, Massachusetts General Hospital Cancer Center, Harvard Medical School, 55 Fruit Street, Boston, MA 02114 USA

**Keywords:** Primary central nervous system lymphoma, High-dose methotrexate, Rituximab, Stem-cell transplant, Whole-brain radiation

## Abstract

Therapeutic options are limited in primary central nervous system lymphoma (PCNSL) with no uniform consensus on optimal management and few published, randomized trials. High-dose methotrexate in combination with other chemotherapeutic agents forms the mainstay of treatment. There hasn’t been much progress beyond high-dose methotrexate in this disease, and although results from trials using high-dose chemotherapy and autologous stem-cell transplant seem promising, these need to be further validated. Moreover, the role of whole brain radiation in the upfront setting remains to be determined. However, international efforts in this direction are underway, with ongoing randomized trials in newly diagnosed PCNSL, more research on the molecular pathogenesis and biomarkers, and the use of novel agents in salvage therapy. There also is emphasis on quality of life parameters and neurocognitive status. Future treatment options should optimize high-efficacy rates while minimizing the risk of neurotoxicity.

## Introduction

Primary central nervous system lymphoma (PCNSL) is an extranodal non-Hodgkin lymphoma (NHL) arising from the brain, spinal cord, eyes or leptomeninges, in the absence of prior or concurrent systemic disease. More than 90 % of PCNSL are diffuse large B-cell lymphomas and are sensitive to chemotherapy and radiation. But relapse rates are high with poor long-term survival compared with other forms of NHL. A minority of the patients does achieve long-term remission (>5 years) with a potential hope for cure, although late relapses have been noted. Moreover, long-term survivors are at a risk of delayed neurotoxicity.

### Epidemiology

PCNSL accounts for 2.2 % of all primary central nervous system tumors, with a median age of 65 years at diagnosis [1]. The annual incidence rate is 0.47 cases per 100,000 person-years [1, 2]. Since 2000, there has been a slight increase in overall incidence, with a dramatic increase in incidence in the elderly, particularly those >75 years of age at diagnosis.

### Diagnosis

The diagnosis of PCNSL can be made by stereotactic brain biopsy, cerebrospinal fluid (CSF) cytopathologic analysis, or by analysis of vitreous aspirate in patients with ocular involvement. The International PCNSL Collaborative Group (IPCG) recommends evaluation for the extent of disease before initiation of therapy [3].

### Prognostic factors

Age and performance status have been consistently identified to be independently associated with prognosis. Two scoring systems (the International Extranodal Lymphoma Study Group (IELSG) scoring system and the Memorial Sloan-Kettering Cancer Center prognostic model) currently exist that help serve as guidelines to determine prognosis and treatment options in newly diagnosed patients, as well as to stratify patients to facilitate comparison across clinical trials [4, 5]. In terms of biomarkers, overexpression of BCL6 has been shown to be associated with better prognosis in most studies except a recent study, which indicated that high BCL6 expression correlated with poor survival [6, 7, 8•].

## Treatment

The treatment of PCNSL involves use of high-dose methotrexate (HD-MTX) in varying doses (1–8 g/m^2^), used as a single agent or in combination with other chemotherapeutic agents and/or radiotherapy. There is no consensus on the dose of HD-MTX or the optimal combination of chemotherapy, neither is there consensus on the role of radiation in combination with methotrexate as first-line therapy. There is no role of surgical resection in treatment of this disease [9].

### Radiotherapy

Historically, PCNSL was treated with whole brain radiation (WBRT), which resulted in high rates of radiographic response but also rapid relapse. WBRT alone is no longer employed for initial treatment. Also, PCNSL is thought to be an infiltrating disease and focal RT is not recommended. A multicenter, phase II trial was conducted in the 1980s in which 41 patients treated with WBRT to 40 Gy plus a 20 Gy boost to the tumor achieved a median overall survival (OS) of 12 months, with majority of the recurrences occurring in the region of the boost [10]. Shibamoto and colleagues reported a median survival of 18 months in a retrospective analysis of 132 patients treated in the 1990s with WBRT monotherapy at different doses [11]. Later studies demonstrated that high doses of methotrexate could achieve therapeutic concentrations in the brain, and when combined with WBRT led to sustained response [12–15]. Most of these studies used high doses of WBRT up to 45 Gy and some included focal boost. However, there was a high incidence of neurotoxicity with this combined modality treatment [16]. This led to studies utilizing lower doses of WBRT. A *post-hoc* analysis of two phase II trials utilizing the same induction chemotherapy, but different doses of WBRT suggested that lowering the dose of consolidation WBRT from 45 to 30.6 Gy led to poor outcomes in patients younger than age 60 years [14]. However, other studies have demonstrated conflicting results. A prospective study showed no neurocognitive decline after consolidation reduced-dose WBRT (23.4 Gy) and cytarabine in patients who had achieved a complete response to induction chemotherapy including HD-MTX [17]. Furthermore, there was no significant cognitive decline up to 24 months after treatment as demonstrated by prospective, neuropsychological testing and six of eight patients were able to return to work [18]. More recently, a retrospective analysis of varying fields and doses of RT in 33 patients who had achieved a complete response to HD-MTX containing regimen indicated that higher WBRT and tumor bed RT doses (>40 Gy) do not improve outcome and are associated with a greater risk of neurotoxicity [19•]. But, delayed neurotoxicity from combined modality treatment especially with advancing age and prolonged progression-free survival (PFS) is well-recognized [20]. Recently, Correa and colleagues identified 50 PCNSL survivors in remission and found that those that were treated with HD-MTX and conventional doses of WBRT had significant neurocognitive dysfunction that interfered with quality of life and >50 % of these patients were not working due to their illness. On the contrary, patients that received HD-MTX alone did not have significant cognitive impairment and had preserved quality of life [21•].

There has been considerable debate whether adding WBRT to methotrexate-based chemotherapy adds to any benefit in terms of PFS or OS. Omuro et al. sought to examine the effects of deferring WBRT. Sixty-four patients younger than age 60 years were treated with a combination of HD-MTX (3 g/m^2^), lomustine, procarbazine, methylprednisolone, and intrathecal methotrexate, cytarabine, and methylprednisolone, of whom 54 % had a complete response and did not receive any further treatment [22]. All other patients underwent WBRT, high-dose chemotherapy followed by autologous stem cell transplant, or maintenance chemotherapy. They found that deferring WBRT affected PFS but not OS. Because salvage WBRT was required, neurotoxicity was delayed but not eliminated. Another retrospective study of 122 patients showed no OS benefit in patients who received consolidation treatment with WBRT compared with those who did not, but higher rates of neurotoxicity were seen in those who received WBRT [23]. Other retrospective studies had similar findings [24, 25].

The German PCNSL study group conducted a phase III trial where patients were randomized to receive HD-MTX–based chemotherapy with or without WBRT [26••]. A total of 551 patients were enrolled of whom 318 were treated per protocol. The authors reported a median OS of 32.4 months (95 % confidence interval (CI), 25.8-39) in the cohort receiving WBRT versus 37.1 months (95 % CI, 27.5-46.7) in the cohort that did not receive WBRT as a part of first-line treatment, with a hazard ratio of 1.06 (95 % CI, 0.8-1.4) and *p* value of 0.71. Although the OS was not statistically different in both groups, the primary hypothesis of non-inferiority of HD-MTX–based regimen over the same chemotherapy followed by WBRT was not proven due to an underpowered study (60 % power) and noninferiority margin of 0.9. Serial neurocognitive testing was not performed, so definitive assessment of neurotoxicity was not possible.

### Chemotherapy

High-dose methotrexate forms the backbone of induction chemotherapy for PCNSL. It is able to achieve cytotoxic levels in the brain parenchyma and CSF [27, 28]. Various studies have used different doses and schedules of HD-MTX, but in general, dose ≥3 g/m^2^ delivered as an initial bolus followed by an infusion over 3 hours, administered every 10 to 21 days is recommended for favorable outcomes and adequate CSF concentrations [25, 29, 30].

There have been various studies using HD-MTX alone or in combination with other cytotoxic agents and/or WBRT. Concern for WBRT-induced neurotoxicity has led to chemotherapy alone trials.

A multicenter, phase II study of single-agent HD-MTX at 8 g/m^2^ given every 14 days as induction therapy followed by monthly maintenance cycles for 12 months in 25 patients yielded an complete response rate (CRR) of 52 % and an overall response rate (ORR) of 74 % with a median PFS of 12.8 months and a median OS of 55.4 months [31, 32]. In another multicenter trial of 37 patients treated with a maximum of six induction cycles of HD-MTX at 8 g/m^2^, the results were not as compelling. The CRR was 30 %, median PFS was 10 months, and the median OS was lower than the other study at 25 months [33]. Studies have demonstrated higher complete response proportions in patients who receive more than six induction cycles of methotrexate-based chemotherapy [17, 31].

In general, combination chemotherapy is thought to achieve higher response rates with longer durability. A pilot and phase II study of 65 patients was conducted using HD-MTX at 5 g/m^2^ and cytarabine in combination with dexamethasone, vincristine, vindesine, ifosfamide, cyclophosphamide, and intraventricular methotrexate, cytarabine, and prednisolone [34]. The investigators reported a CRR of 61 %, median PFS of 21 months and median OS of 50 months. At a median follow up of 100 months, 32 % of all patients and 57 % of patients ≤60 years of age were still alive [35]. However, there was a fair amount of toxicity associated with this regimen; 9 % of patients died and 19 % developed infections of the Ommaya reservoir. Interestingly, serial neuropsychologic testing demonstrated no evidence of cognitive decline in the majority of the patients. Due to the high rate of Ommaya reservoir infections in the prior trial, the same group conducted another phase II trial with the same systemic regimen without the intraventricular treatment in 18 patients [36]. The CRR was 53 %, the median duration of response was only 10 months, and median PFS was 8 months in all patients, as a result of which the trial was terminated early. The results of this study seem to support a role intraventricular chemotherapy. However, a retrospective study demonstrated no benefit in OS, PFS or neurotoxicity of adding intrathecal methotrexate to a HD-MTX–based regimen [37]. Similar results were found in another retrospective analysis of 69 PCNSL patients with no evidence of positive or suspicious CSF cytology, of whom 39 received HD-MTX–based chemotherapy (1–3 g/m^2^ depending on age) and intrathecal methotrexate, cytarabine, and methylprednisolone, and the other 30 received the same systemic chemotherapy with omission of intrathecal treatment [38•]. The authors found no significant difference in response rates, patterns of relapse, and OS regardless of intrathecal treatment. The median PFS was 28.7 months (95 % CI, 11.1-40.1) in the group receiving intrathecal prophylaxis versus 9.9 months (95 % CI, 5.6-27.4) in the other group, with a *p* value of 0.0518.

Ferreri and colleagues reported the results from the first successfully completed randomized, phase II trial in PCNSL where 79 patients were treated with single-agent HD-MTX (3.5 g/m^2^ every 21 days) versus HD-MTX and cytarabine [15]. All patients underwent WBRT after induction chemotherapy. The authors reported improved efficacy and activity with the addition of cytarabine to methotrexate and demonstrated that combination chemotherapy is better than methotrexate monotherapy at this dose and schedule. CRR was 46 % in the cytarabine plus methotrexate arm versus 18 % in methotrexate monotherapy arm, and 3-year OS was 46 % and 32 %, respectively.

Intra-arterial delivery of methotrexate in conjunction with blood–brain barrier disruption has been studied and reported in a series of 149 patients from multiple institutions [39]. The authors reported an ORR and CRR of 82 % and 58 %, respectively, with a median PFS and OS of 1.8 and 3.1 years, respectively. Toxicities unique to this method of delivery included peri-procedural seizures in 33.6 %, carotid or vertebral artery injury in 10.7 %, and strokes in 7.4 % of the patients.

More recently, rituximab, a chimeric monoclonal antibody targeting CD20 antigen on B-lymphocytes, is being incorporated in combination regimens. When administered in doses of 375–800 mg/m^2^, CSF levels from 0.1 % to 4.4 % of serum levels have been noted on pharmacokinetic studies [17, 40].

Shah et al. demonstrated a CRR of 78 % after 7 cycles of HD-MTX at 3.5 g/m^2^ in combination with rituximab, procarbazine, and vincristine [17]. All patients underwent WBRT (23.4 vs. 45 Gy depending on response to induction) followed by cytarabine. The estimated 2-year PFS and OS was 57 % and 67 % after a median follow-up of 37 months.

Rubenstein and colleagues studied the effects of a 2-step dose-intensive immunochemotherapeutic regimen in a multicenter cooperative group setting [8•]. In this trial, 44 patients were treated with induction chemotherapy consisting of HD-MTX at 8 g/m^2^ (day 1), rituximab at 375 mg/m^2^ (day 3), and temozolomide at 150 mg/m^2^ (days 7–11). Each of the drugs in the MTR induction regimen has been studied as single agents with demonstrated activity in PCNSL. MTR induction was followed by consolidation chemotherapy that included intravenous etoposide 5 mg/kg as a continuous infusion over 96 hours (total 40 mg/kg) and cytarabine at 2 g/m^2^ every 12 hours for 8 doses. The CRR on induction chemotherapy was 66 %. With a median follow-up of 4.9 years, the 2-year PFS was 57 %, the median PFS was 4 years, and the median OS was not reached. Major toxicity was grade 4 neutropenia and thrombocytopenia seen in approximately 50 % of the patients, and the majority of these episodes were after consolidation chemotherapy. There was one treatment-related death. These results are encouraging and are comparable to any regimen that *includes* WBRT. Moreover, in this study median PFS was similar for older (>60 years of age) and younger patients. This is an interesting finding as prior studies have shown that patients older than 60 years of age have worse outcomes [5]. The authors also reported shorter PFS in patients that delayed induction chemotherapy beyond 1 month of diagnosis compared to those that did not (3-year PFS of 20 % vs. 59 %, *p* = 0.05).

### High-dose chemotherapy and stem cell transplantation

Chemotherapy and radiation have high efficacy rates in PCNSL, but responses are not durable. Moreover, there is always the issue of neurotoxicity from radiation. There has been emerging data on the role of high-dose chemotherapy (HDCT) followed by autologous stem cell transplantation (ASCT) as first-line therapy for consolidation in PCNSL. Abrey et al. treated 28 patients with HD-MTX and cytarabine for induction followed by treatment with carmustine, etoposide, cytarabine, melphalan (BEAM), and ASCT [41]. The results were disappointing with a median PFS of 5.6 months for all patients and 9.3 months for those who underwent ASCT. Subsequently, a multicenter, phase II trial was conducted in which 25 patients <60 years of age received induction chemotherapy with HD-MTX, etoposide, carmustine, and methylprednisolone, then ifosfamide and cytarabine, followed by BEAM and ASCT, after which patients received WBRT [42]. This study demonstrated better results with a median PFS of 40 months. More recently, conditioning regimens have included thiotepa and results have been more encouraging, likely due to better CNS penetration. In a phase II trial, 23 patients were treated with HD-MTX followed by high-dose thiotepa/busulfan and ASCT, and response-adapted WBRT. The median PFS was 17 months [43]. On long-term follow-up 10 years after initiation of study, the OS rate was 35 %; six patients remained in complete remission, and the majority of the patients who did not receive WBRT had no evidence of neurotoxicity [44•]. Illerhaus and colleagues treated 43 patients with one of two HD-MTX–based induction regimens followed by high-dose carmustine/thiotepa and ASCT with/without WBRT in two prospective trials [45, 46]. They reported a 5-year OS of 70 % and 5-year PFS of 67 % [47]. In a single-center study of 11 patients treated with HD-MTX and cytarabine, followed by busulfan, cyclophosphamide, etoposide, and ASCT, the median PFS was 15 months [48]. Recently, Illerhaus et al. reported the results of a multicenter study in which 79 patients were treated with induction HD-MTX, cytarabine, rituximab and thiotepa, followed by carmustine/thiotepa conditioning before ASCT, with an ORR of 91 % and 2-year OS 87 % [49•]. Five patients died from the treatment. A retrospective analysis of 105 patients treated with HDCT followed by ASCT with/without WBRT demonstrated 5-year OS of 79 % and median PFS and OS of 85 and 121 months, respectively [50•]. Based on the results of these phase II trials, this mode of consolidative treatment seems very promising. The toxicities, mostly cytopenias, are manageable. There are two ongoing, multicenter, randomized trials comparing the efficacy of consolidative HDCT and ASCT versus chemotherapy or WBRT (Table 1).

**Table 1:** Randomized trials in primary central nervous system lymphoma (PCNSL)

Induction		Consolidation	
Completed trials		Completed trials	
1	Ferrerri *et al.* (IESLG) *Ref.* 15 Phase II, n = 79, age 18–75y **MTX + HD-Ara-C vs. MTX**; (followed by WBRT in all), ORR: 69 % vs. 40 % (*p* = 0.009) 3y, PFS: 20 % vs. 11 % 3y, OS: 46 % vs. 32 % (*p* = 0.07)	1	Thiel *et al*. (G-PCNSL-SG-1) *Ref.* 26 Phase III, n = 318, age > 18y (MTX ± ifosfamide) **± WBRT, ** mOS: 32.4 vs. 37.1 mo (*p* = 0.71) , mPFS: 18.3 vs. 11.9 mo (*p* = 0.14)
2	Omuro *et al.* (ANOCEF-GOELAMS) *Ref.* 61 Phase II, n = 95, age ≥ 60y **MPV-A vs MT, ** ORR: 82 % vs. 71 % (*p* = 0.23) , mPFS: 9.5 vs. 6.1 mo (*p* = 0.6) , mOS: 31 vs. 13.8 mo (*p* = 0.2)		
Ongoing trials		Ongoing trials	
1	IESLG - NCT01011920 Phase II **AM vs. AMR vs. AMRT ** (followed by WBRT vs. HDCT+ASCT)	1	IESLG - NCT01011920 Phase II (HD-MTX+Ara-C±R+T) followed by **WBRT vs. HDCT (BT)+ASCT**
2	HOVON/ALLG - NTR2427 (Netherlands trial register) Phase III, open-label **MBVP vs. R-MBVP ** (followed by Ara-C+WBRT in all)	2	ANOCEF-GOELAMS - NCT00863460 Phase II (R-MBVP, R-aracytine) followed by **WBRT vs. HDCT (TBC)+ASCT**
		3	RTOG - NCT01399372 Phase II (RMPV-A) **± low-dose WBRT**
		4	CALGB-NCI - NCT01511562 Phase II (MTR-A) followed by **HDCT (BT)+ASCT vs.** **Ara-C+etoposide**

^*^MTX, high-dose methotrexate; HD-AraC, high-dose cytarabine; WBRT, whole brain radiation therapy; ORR, objective response rate; PFS, progression-free survival; OS, overall survival; m, median; MPV-A, high-dose methotrexate, procarbazine, vincristine, cytarabine; MT, high-dose methotrexate, temozolomide; AM, cytarabine, high-dose methotrexate; AMR, cytarabine, high-dose methotrexate, rituximab; AMRT, cytarabine, high-dose methotrexate, rituximab, thiotepa; HDCT, high-dose chemotherapy; BT, BCNU(carmustine), thiotepa; R-MBVP, rituximab, high-dose methotrexate, BCNU, etoposide or teniposide, prednisone; TBC, thiotepa, BCNU, cyclophosphamide; RMT-A, rituximab, high-dose methotrexate, temozolomide, cytarabine

### Treatment in the elderly

The elderly account for more than half of the patients with PCNSL [51]. The risk of neurotoxicity is the highest in this population, and in general, chemotherapy alone is a reasonable option. In a study using a HD-MTX–based regimen and WBRT, almost 90 % of patients >60 years of age developed delayed neurotoxicity and died from related complications, rather than recurrent disease [52]. On the other hand, almost 50 % of older patients who deferred WBRT died of progressive disease. Thus, deferring WBRT in older patients reduced treatment-related neurotoxicity and did not compromise OS. Various studies have indicated that HD-MTX at doses of 3.5-8 g/m^2^ is well tolerated in these patients with minimal grade 3 or 4 renal and hematological toxicity [53–55]. Zhu and colleagues reported a series of 31 patients ≥70 years of age who were treated with a median of eight cycles of single-agent HD-MTX at 3.5-8 g/m^2^ and demonstrated an ORR of 96.7 % with a CRR of 60 % [54]. The median PFS was 7.1 months and median OS was 37 months. In a retrospective study of 24 patients ≥80 years of age, 23 were treated with HD-MTX–based regimen without significant renal toxicity despite low creatinine clearance at baseline and were found to have a 2-year OS of 33 % [56].

Combination chemotherapy has been tried successfully in older patients and found to be relatively well-tolerated. A phase II trial was conducted in 23 patients >60 years of age who received HD-MTX at 3 g/m^2^ (days 1, 10, 20) and temozolomide at 100 mg/m^2^ (days 1–5) followed by up to 5 maintenance monthly cycles of HD-MTX and temozolomide in responders [57]. A CR was seen in 55 % of the patients, and median PFS and OS were 8 and 35 months, respectively. A multicenter phase II trial of up to six cycles of HD-MTX (5 g/m^2^), lomustine, vincristine, methylprednisolone, and intrathecal methotrexate and cytarabine in 50 patients with median age of 72 years (>60 years) resulted in a CRR of 42 %, with median PFS and OS of 10.6 and 14.3 months [58]. Illerhaus and colleagues evaluated the effects of three 45-day cycles of HD-MTX at 3 g/m^2^ (days 1, 15, 30) with procarbazine at 60 mg/m^2^ (days 1–10) and lomustine at 110 mg/m^2^ (day1) in a phase II trial with 30 patients ≥65 years of age [59]. CRR was 44.4 %, and median PFS and OS were 5.9 and 15.4 months, respectively. Subsequently, the same group conducted another trial with the addition of rituximab to the same regimen [60]. In 28 patients, the CRR was 64 %, with a median PFS and OS of 16 and 17.5 months respectively, and 3-year PFS and OS rate of 31 %. Median OS was remarkably better in patients <80 years of age at 29 months, and 4.3 months in those older. There were two treatment-related deaths. The ANOCEF-GOELAMS study was a multicenter, randomized, phase II trial of chemotherapy alone in elderly patients with PCNSL [61•]. In this study, 98 patients were randomized to receive three cycles of either MPV-A (methotrexate 3.5 g/m^2^, days 1 and 15; procarbazine 100 mg/m^2^, days 1–7; vincristine 1.4 mg/m^2^, days 1 and 15) or MT (methotrexate 3.5 g/m^2^, days 1 and 15; temozolomide 100–150 mg/m^2^, days 1–5, 15–19) with one additional cycle of cytarabine (3 g/m^2^/day for 2 consecutive days) in the MPV arm only. While there were trends favoring the MPV-A regimen over the MT regimen with respect to complete response rate, PFS and OS, none of these differences reached statistical difference. Recently published data suggest that the addition of rituximab to both MPV and MT increased the radiographic response rate. Both of these chemotherapy regimens are options in elderly PCNSL patients.

### Salvage treatment

Despite high response rates seen with initial HD-MTX based treatments, many patients with PCNSL relapse. Moreover, there is a small subset of patients that have methotrexate-refractory disease. Prognosis of progressive or relapsed PCNSL is poor with a limited number of prospective studies for guidance on management. Rechallenge with HD-MTX has been shown to be effective in patients that had previously responded to it. In a multicenter retrospective review of 22 patients, 91 % had a radiographic response to first salvage treatment with HD-MTX and 100 % to second salvage [62]. The median OS from first salvage was 61.9 months.

Pemetrexed, which is similar to methotrexate with antifolate activity, has been shown to been efficacious in recurrent or refractory PCNSL. In a trial of 11 patients who were treated with a median of 5 cycles of pemetrexed at 900 mg/m^2^ administered every 3 weeks, there was a ORR of 55 % with 6-month PFS of 45 % [63•].

Rituximab and temozolomide have been studied in combination as well as single agents in relapsed PCNSL. A retrospective review of 17 patients with single-agent temozolomide demonstrated a median OS of 6.7 months [64]. A prospective study of 36 patients treated with temozolomide reported a ORR and 1-year OS of 31 % [65]. In a multicenter pilot study of single agent rituximab in 12 patients, the ORR was 31 % with a median OS of 20.9 months [66•]. While prior retrospective studies of combination rituximab and temozolomide demonstrated promising results of ORR of 53-100 % and 1-year OS of 55 %, a multicenter phase II trial failed to reproduce the results with early closure of the trial for a poor response rate of 14 % and median PFS of 7 weeks [67–69]. However, this may have been due to patient selection bias. Given this regimen is very well tolerated, it is recommended in a selected population of patients.

Topotecan also has been found to be useful in salvage treatment with response rates of 33-40 % and median OS of 8.4 and 35 months in two different studies [70, 71]. Other salvage treatments have included combination of etoposide, ifosfamide, and cytarabine, and procarbazine, lomustine, and vincristine with response rates of 37 % and 86 %, respectively, and 1-year OS of 41 % and 57 %, respectively [72, 73]. Rubenstein and Treseler successfully treated a patient with intraocular lymphoma with lenalidomide, an immunomodulating drug (IMiD) [74].

Intraventricular rituximab has been studied by itself and in combination with intraventricular methotrexate in phase I trials [75, 76]. Fourteen patients were treated with intraventricular rituximab and methotrexate, of whom 43 % had a CR in CSF and/or brain [76].

The most impressive results are from a phase II trial conducted by Soussain and colleagues in 43 patients who were treated with initial high-dose cytarabine and etoposide followed by HDCT with thiotepa, busulfan, cyclophosphamide, and ASCT [77]. In this study, 27 patients underwent transplantation, of whom 26 had a CR. The median PFS and OS in these 27 patients were 41.1 and 58.6 months, respectively. Kasenda et al. demonstrated that a second autotransplantation is successful as salvage treatment in patients who have previously undergone HDCT and ASCT [78].

Allogeneic peripheral blood stem cell transplantation (allo PBSCT) has been found to be successful in patients with leukemia/lymphoma with CNS involvement [79–82]. There is one case report in the literature in which the authors reported a patient with PCSNL who was in complete remission 3 months after the transplantation and remained so for 30 months [83].

Finally, WBRT in patients who have not received it as a part of initial treatment is an effective option, although there is certainly a risk of neurotoxicity. Many clinicians reserve this for those with chemotherapy-refractory disease in whom the disease has progressed through chemotherapy at initial treatment or at relapse. In a series of 27 patients treated with WBRT (median dose 36 Gy), 74 % achieved a radiographic response and median OS was 10.6 months [84]. Late neurotoxicity rates of 15 % were noted at doses >36 Gy. Another study in 48 patients treated with a median dose of 40 Gy, reported an ORR of 79 % with a median OS of 16 months and treatment-related neurotoxicity in 22 % of patients [85].

### Trials in progress

Although randomized clinical trials have been lacking in the field of PCNSL, there are now several clinical trials through cooperative groups investigating various questions regarding optimal treatment strategies (Table 1). These studies are designed to examine the optimal induction regimen, the role of rituximab in induction, the benefit of consolidative reduced-dose radiation, as well as comparing high-dose chemotherapy to myeloablative chemotherapy for consolidation. There are currently two trials in progress that are comparing consolidative WBRT versus HDCT and ASCT, both utilizing different conditioning regimens prior to ASCT. Some of these studies also include correlative biomarkers and imaging components as well as assessment of neurocognitive and quality of life outcomes assessment.

In terms of salvage therapy, novel agents are currently being tested after their success in systemic B-cell lymphoma. Currently, there are ongoing trials utilizing the combination of lenalidomide and rituximab (NCT01542918), single-agent pomalidomide (NCT01722305), and single-agent temsirolimus (NCT00942747) in treatment of relapsed PCNSL.
